# Distribution of Antibiotic Resistance Genes in the Saliva of Healthy Omnivores, Ovo-Lacto-Vegetarians, and Vegans

**DOI:** 10.3390/genes11091088

**Published:** 2020-09-18

**Authors:** Vesna Milanović, Lucia Aquilanti, Stefano Tavoletti, Cristiana Garofalo, Andrea Osimani, Francesca De Filippis, Danilo Ercolini, Ilario Ferrocino, Raffaella Di Cagno, Silvia Turroni, Camilla Lazzi, Nicoletta Pellegrini, Francesca Clementi

**Affiliations:** 1 Department of Agricultural, Food and Environmental Sciences (D3A), Università Politecnica delle Marche, 60131 Ancona, Italy; v.milanovic@staff.univpm.it (V.M.); s.tavoletti@staff.univpm.it (S.T.); c.garofalo@staff.univpm.it (C.G.); a.osimani@staff.univpm.it (A.O.); f.clementi@staff.univpm.it (F.C.); 2 Department of Agricultural Sciences, Division of Microbiology, University of Naples Federico II, 80055 Portici (NA), Italy; francesca.defilippis@unina.it (F.D.F.); ercolini@unina.it (D.E.); 3 Task Force on Microbiome Studies, University of Naples Federico II, 80055 Portici (NA), Italy; 4 Department of Agricultural, Forest and Food Science (DISAFA), University of Turin, 10095 Grugliasco (TO), Italy; ilario.ferrocino@unito.it; 5 Faculty of Science and Technology, Libera Università di Bolzano, 39100 Bolzano, Italy; raffaella.dicagno@unibz.it; 6 Department of Pharmacy and Biotechnology, Alma Mater Studiorum, University of Bologna, 40126 Bologna, Italy; silvia.turroni@unibo.it; 7 Department of Food Science, University of Parma, 43121 Parma, Italy; camilla.lazzi@unipr.it; 8 Department of Agricultural, Food, Environmental and Animal Sciences, University of Udine, 33100 Udine, Italy; nicoletta.pellegrini@unipr.it

**Keywords:** antibiotic resistance genes, dietary habits, human saliva, human salivary resistome, oral cavity

## Abstract

Food consumption allows the entrance of bacteria and their antibiotic resistance (AR) genes into the human oral cavity. To date, very few studies have examined the influence of diet on the composition of the salivary microbiota, and even fewer investigations have specifically aimed to assess the impact of different long-term diets on the salivary resistome. In this study, the saliva of 144 healthy omnivores, ovo-lacto-vegetarians, and vegans were screened by nested PCR for the occurrence of 12 genes conferring resistance to tetracyclines, macrolide-lincosamide-streptogramin B, vancomycin, and β-lactams. The *tet*(W), *tet*(M), and *erm*(B) genes occurred with the highest frequencies. Overall, no effect of diet on AR gene distribution was seen. Some differences emerged at the recruiting site level, such as the higher frequency of *erm*(C) in the saliva of the ovo-lacto-vegetarians and omnivores from Bologna and Turin, respectively, and the higher occurrence of *tet*(K) in the saliva of the omnivores from Bologna. A correlation of the intake of milk and cheese with the abundance of *tet*(K) and *erm*(C) genes was seen. Finally, when the occurrence of the 12 AR genes was evaluated along with geographical location, age, and sex as sources of variability, high similarity among the 144 volunteers was seen.

## 1. Introduction

The human oral cavity hosts a heterogeneous microbiota that includes commensal bacteria with a fundamental role in the maintenance of both oral and systemic health [1]. The composition of this microbiota and especially of bacterial communities associated with endodontic and periodontal infections has been investigated in several studies. Thus far, a plethora of taxa are known to colonize the human oral cavity in either a suspended (planktonic state) or attached (biofilm) state [2,3,4,5]. In the past decade, research efforts have focused on determining those factors modulating the complexity of this peculiar microbiota, such as geographical location [6], clinical conditions [7,8], obesity [9], and even dietary habits and intakes [10,11,12].

Dietary intake undoubtedly represents one of the main routes for microorganisms and their genes to enter the human body [13]. In the mouth, which constitutes the principal entry point for a wide variety of bacteria, food is chewed, mixed with saliva, propelled through the esophagus by gravity and then mobilized to the stomach and intestine by peristalsis. Along this path, microorganisms contained in the food bolus intercept the commensal bacteria that colonize the various gastrointestinal-associated habitats and potentially exchange genetic material with this resident microbiota [14]. Horizontal gene transfer between foodborne microorganisms and oral residential bacteria by conjugation [15] and the transfer of conjugative transposon-encoded antibiotic resistance (AR) genes in oral community biofilms by transformation [16] have already been demonstrated. Even naked DNA has been reported to transform naturally competent bacteria that stably colonized the oral cavity [17]. Among mobilized genes, those conferring resistance to antibiotics are commonly exchanged through diverse mechanisms such as transformation, transduction, and conjugation [18].

In recent years, numerous retail foods have been found to harbor antibiotic-resistant pathogens [19]. However, these microorganisms do not represent a significant source for the horizontal transmission of AR genes given their relatively low numbers in food and food-related ecosystems. More recently, numerous AR genes have been found in commensal bacteria isolated from conventional retail foods, including ready-to-eat products [15], thus clearly suggesting that the human population is continuously exposed to antibiotic-resistant microorganisms through daily food intake [20]. In addition, multiple research studies have demonstrated the prevalence of antibiotic-resistant bacteria in the oral ecosystems of healthy subjects not previously exposed to antibiotics, such as neonates and breast-fed infants [21,22], thus indicating that the emergence and dissemination of AR genes can be independent of the selective pressure exerted by antibiotics [23,24,25].

Hence, the question is whether different food habits might affect the abundance and diversity of transferable AR genes in the human oral cavity. To address this issue, saliva collected from a large cohort of healthy volunteers who were enrolled at four Italian locations (Bari, Turin, Bologna, and Parma) and who were following a long-term omnivore, ovo-lacto-vegetarian, or vegan diet were screened by PCR and nested PCR for the occurrence of 12 genes that were selected from among those conferring resistance to four classes of antibiotics conventionally used in both agriculture (e.g., animal husbandry and crop production) and clinical practice, namely, tetracyclines, macrolide-lincosamide-streptogramin B (MLS_B_), vancomycin, and β-lactams. More specifically, the 12 target genes were chosen from among those frequently detected in food commensals, mainly represented by lactic acid bacteria (LAB) [26], and human pathogens isolated from foods and food producing animals [27] and those showing a high-risk potential to be introduced into human pathogens [28].

The results of the molecular screening were statistically analyzed to define the effect of diet on the frequency and distribution of the AR determinants under study. In addition, the effects of geographical location, sex, and age of the volunteers on the occurrence of the 12 selected AR genes were also investigated.

The present research represents a follow-up of a previous study aimed at evaluating the effects of long-term omnivore, ovo-lacto vegetarian, and vegan diets on the occurrence of the same 12 target genes in the human gut of the same cohort of subjects herein considered [29]. In this regard, Carr et al. [30] have very recently pointed out the necessity for extensive AR surveillance studies across different body sites, including the gut and the oral cavity. Interestingly, the analyses done on the feces showed a low effect of diet and a high impact of the geographical location on the AR gene distribution [29]. On the other hand, a positive correlation of the consumption of eggs, milk and cheese of animal origin with the abundance of *tet*(K) was observed, while the occurrence of *vanB* was positively correlated with the regular intake of poultry meat, eggs, fish, and seafood.

Finally, hints from the comparative evaluation of data collected from the saliva samples herein analyzed and feces from the same cohort of volunteers [29] were outlined.

## 2. Materials and Methods

### 2.1. Recruitment of Healthy Volunteers with Omnivore, Ovo-Lacto-Vegetarian and Vegan Diets

A cohort of 144 healthy nonsmoking volunteers (85 females and 59 males) with body mass index (BMI) > 18 (22 ± 2.3) and age between 18 and 59 years (38 ± 9.3) were recruited from February to July 2013 at four Italian locations, namely, Bari (BA, 39 volunteers), Turin (TO, 36 volunteers), Bologna (BO, 36 volunteers) and Parma (PA, 33 volunteers). The recruitment of volunteers, carried out between February and July 2013, was aimed at investigating the effects of long-term omnivore, ovo-lacto-vegetarian, and vegan diets on the human gut microbiota (http://clinicaltrials.gov/ct2/show/NCT02118857). The cohort of subjects included 48 omnivores, 48 ovo-lacto-vegetarians and 48 vegans with stable dietary habits (≥1 year). A vegan diet was assumed for those subjects who did not consume any food of animal origin, including eggs, milk and milk-based products, whereas an ovo-lacto vegetarian diet was assumed for those subjects who did not consume any meat, fish, and seafood. The detailed list of recruited volunteers, each identified with an anonymous code, diet, age, sex and geographical location, is reported in Table S1. As detailed in similar studies [10,29,31,32,33], the exclusion criteria for recruiting volunteers were: (i) age < 18 and >60 years; (ii) vegan, ovo-lacto vegetarian and omnivore diets followed for less than 1 year; (iii) exposure to antibiotics, probiotics or drugs in the previous 3 months; (iv) surgical operations during the previous 3 months; (v) pathologies such as Crohn’s disease, chronic ulcerative colitis, bacterial overgrowth syndrome, constipation, celiac disease, irritable bowel syndrome, diabetes mellitus, cardiovascular or cerebrovascular diseases, cancer, neurodegenerative disease, rheumatoid arthritis, and allergies; and vi) pregnancy and breastfeeding.

All participants were informed about the aims of the study and provided informed written consent. The study was approved by the Ethics Committee of (i) Azienda Sanitaria Locale (Bari) (protocol N.1050), (ii) Azienda Ospedaliera Universitaria of Bologna (protocol N.0018396), (iii) Province of Parma (protocol N.22884) and (iv) University of Torino (protocol N.1/2013/C).

All methods were carried out in accordance with relevant guidelines and regulations.

### 2.2. Saliva Sample Collection and DNA Extraction

Stimulated whole saliva samples (approximately 5 mL) were supplied weekly by the 144 volunteers in sterile empty containers (50 mL) over a time span of three weeks, once per week. The samples were collected after about 2 h fasting from tooth-brushing. The procedures used for collection, handling, and storage of the saliva samples have previously been detailed [9,10]. During the 3-week sampling period, the enrolled volunteers were asked to fill in weighed food diaries in which they daily recorded all food and beverages consumed [10,29].

Triplicate saliva samples were pooled together to limit intraindividual variability prior to DNA extraction with a BiOstic Bacteremia DNA Isolation Kit (MoBIO Laboratories, Inc., Carlsbad, CA, USA) as previously described [10].

### 2.3. PCR and Nested PCR Screening of the Saliva Samples

For each pooled sample, approximately 10 ng of DNA, which was quantified using a spectrophotometer (NanoDrop 1000, Thermo Scientific, Milano, Italy), was amplified in a 25 μL reaction volume using a MyCycler thermal cycler (Bio-Rad Laboratories, Hercules, CA, USA). In the case of a negative result, 2 μL of the first PCR product was reamplified using external primers. The primer pair sequences, amplification conditions, thermal cycling profiles, and detection limits of the PCR and nested PCR assays used for the detection of *tet*(M), *tet*(W), *tet*(O), *tet*(S), *tet*(K), *erm*(A), *erm*(B), *erm*(C), *vanA*, *vanB*, *blaZ,* and *mecA* have previously been described [29,34]. In all PCR and nested PCR assays, both positive and negative quality controls were systematically processed to ensure data integrity, accuracy, and reproducibility and to identify errors and/or omissions occurring during processing [29]. Given the high sensitivity of the nested PCR protocols, which showed limits of detection up to 7 times lower than those of the corresponding PCR protocols [34], all PCR mixtures for amplification were prepared at a PCR workstation equipped with UV light, which is intended to prevent contamination by air UV-irradiation and nucleic acid denaturation. In addition, physically separate work areas were dedicated to pre- and post-PCR activities. Aliquots (5 μL) of each PCR and nested PCR product were analyzed by electrophoresis. The specificity of the amplification reactions was checked by sequencing randomly selected amplicons [29]. Due to the high sensitivity of the amplification systems used, several strategies were adopted to limit contamination events due to amplicon carryover, as previously described [29]. The presence or absence of each AR gene as revealed by PCR and nested PCR runs was converted into a data file that was applied for statistical analysis.

### 2.4. Statistical Analysis

#### 2.4.1. Pearson’s Chi Square (χ^2^) Tests

Data recorded from the 144 healthy volunteers in the weighed food diaries were first averaged to obtain the daily consumption of all foods and beverages. Then foods and beverages were grouped into 11 arbitrarily defined food categories, namely, “red meat”, “white meat”, “preserved meat”, “cheese”, “milk and yogurt from animal sources”, “milk from vegetable sources and its derivatives”, “fish and seafood”, “eggs”, “fruit”, “vegetables”, and “pulses”. For each volunteer and food category, the average daily intake (expressed as g or mL day^−1^) ± standard deviation was calculated.

For the PCR and nested PCR data, for each AR gene of interest, the absolute detection frequency was calculated as the ratio between samples that were PCR-positive and the total number of PCR-processed samples. A contingency analysis based on the log likelihood ratio test (α = 0.05) was used to test the influence of diet on the occurrence of AR genes.

The relationships between AR genes (e.g., presence vs. absence) and volunteer age, sex, and daily food consumption from the 11 arbitrarily defined categories were tested by the χ^2^ test (α = 0.05, 1 degree of freedom) with 2 × 2 contingency tables that were obtained by grouping the volunteers into two classes, A and B. For the variable “age”, class A included 78 volunteers aged ≥37 years whereas class B included 66 volunteers aged <37. Regarding the variables “fruit”, “vegetables” and “pulses”, class A included 72 volunteers eating ≥267 g day^−1^ of fruit, 73 volunteers eating ≥379 g day^−1^ of vegetables, and 74 volunteers eating >57 g day^−1^ of pulses, whereas class B included 72 volunteers eating <267 g day^−1^ of fruit, 71 volunteers eating <379 g day^−1^ of vegetables, and 70 volunteers eating <57 g day^−1^ of pulses. For the remaining variables, classes A and B included volunteers eating or not eating from specific food categories.

#### 2.4.2. Principal Component Analysis (PCA)

The effect of dietary habits and geographical sites on AR gene frequencies was visualized by principal component analysis (PCA) using NTSYS 2.02i software (Applied Biostatistics Inc., Exeter Software, Setauket, New York, NY, USA). Data from PCR and nested PCR, expressed as the presence or absence of each AR gene under study, were converted into a 1 (presence)/0 (absence) table including the 144 healthy volunteers and 12 AR genes under study. This dataset was used to build a table with the relative frequencies of positive volunteers characterizing the 12 groups (diets x sites), and a Pearson correlation matrix was obtained to conduct the PCA. Eigenvalues and eigenvectors were analyzed to identify the most important principal components and relative importance of AR genes within each principal component, respectively. The results were graphically summarized to identify possible grouping patterns among the 12 groups evaluated.

Moreover, a χ^2^ analysis was performed separately for each geographical location to detect the AR genes responsible for the differences among the three diets. The PCA results guided the choice of the most interesting orthogonal contrasts to be tested.

## 3. Results

### 3.1. Cohort of Heathy Volunteers Following Long-Term Omnivore, Ovo-Lacto-Vegetarian and Vegan Diets

Table 1 shows the daily average consumption (expressed as g day^−1^) by the three groups of volunteers (e.g., omnivores, ovo-lacto-vegetarians, and vegans) of 11 potentially high-risk foods for introduction of AR microorganisms and their genes into the human oral cavity. Detailed data on the daily average consumption of single food types by each volunteer enrolled in the project have previously been reported by Milanović et al. [29]. The latter study aimed at assessing the impact of the three long-term diets on the gut resistome of the same cohort of subjects; significant diet-dependent variability in the intake of single food types by the three groups of volunteers was seen. In addition to the expected differences between omnivores, ovo-lacto-vegetarians and vegans, even geographically dependent dietary habits reflecting local practices and traditions were revealed.

### 3.2. PCR and Nested PCR

The results of the PCR-based screening are shown in Table 2. For *erm*(B), *tet*(M), and *tet*(W), more than 50% of the processed samples were positive in the first PCR runs, whereas for *erm*(A), *vanA* and *tet*(O), a relatively low number of samples were positive only after the second amplification reaction. Moreover, two genes, namely, *vanB* and *mecA,* remained undetectable by both PCR and nested PCR.

**Figure 1:**
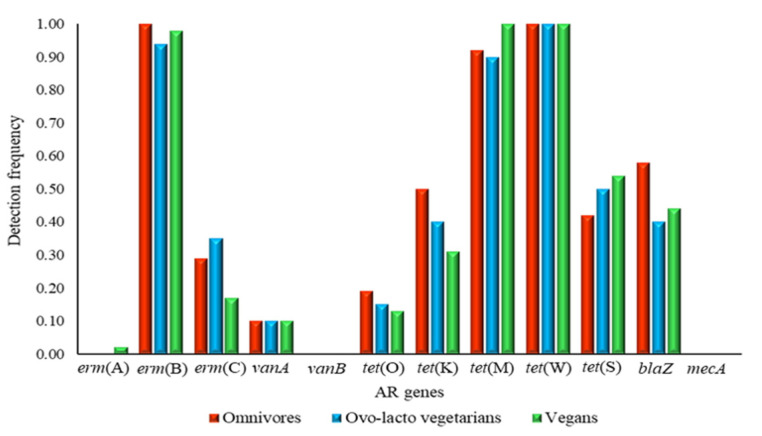
Detection frequency of 12 target antibiotic resistance (AR) genes in the saliva of volunteers following an omnivore, ovo-lacto vegetarian and vegan diet.

The relative detection frequencies of the target AR genes within each dietary group of volunteers are shown in Figure 1. Overall, the genes coding for resistance to tetracyclines and macrolide-lincosamide-streptogramin B were the most frequently detected. Among the genes conferring resistance to tetracyclines, *tet*(W) was clearly the most abundant, since it was detected in all samples analyzed (frequency = 1.00). It was followed by *tet*(M), occurring at frequencies ranging from 0.90 (ovo-lacto-vegetarians) to 1.00 (vegans); *tet*(S), occurring at frequencies ranging from 0.42 (omnivores) to 0.54 (vegans); and *tet*(K), occurring at frequencies between 0.31 (vegans) and 0.50 (omnivores). Among the *tet* genes, *tet*(O) was the least abundant and was detected with frequencies ranging from 0.13 (vegans) to 0.19 (omnivores).

Among the *erm* genes, *erm*(B) clearly dominated; it was detected at frequencies between 0.94 (ovo-lacto-vegetarians) to 1.00 (omnivores), and was followed by *erm*(C), which occurred at frequencies ranging from 0.17 (vegans) to 0.35 (ovo-lacto-vegetarians). The *erm*(A) gene was found in only one saliva sample, which was collected from a vegan.

A very limited occurrence of the two genes conferring high levels of resistance to vancomycin was observed with no positive samples for *vanB*, while *vanA* was detected at a very low frequency (0.10) in all three dietary groups (vegans, ovo-lacto-vegetarians and omnivores).

Finally, for the genes coding for resistance to β-lactams, *bla*Z was detected with frequencies ranging from 0.40 (ovo-lacto-vegetarians) to 0.58 (omnivores), whereas no samples were positive for *mec*A.

### 3.3. χ^2^ Tests

**Figure 2:**
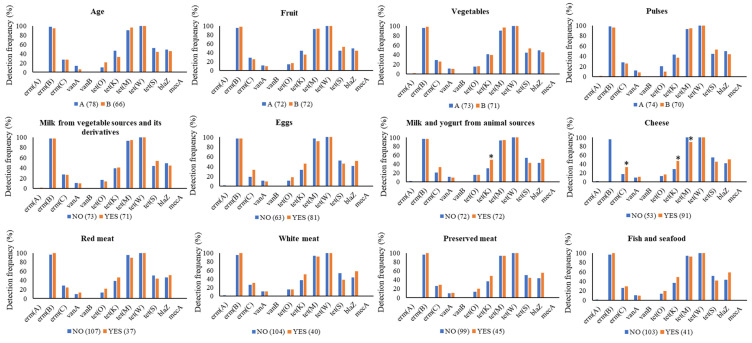
The effect of age and average daily consumption of eleven food categories on detection frequency (%) of the 12 AR genes under study according to the results of Chi square (*X*^2^) test. Volunteers were grouped into two classes for each category, as follows: “age”: class A (age < 37 years old), class B (age ≥ 37 years old); “fruit”: class A (<267 g day^−1^), class B (≥267 g day^−1^); “vegetables”: class A (<379 g day^−1^), class B (≥379 g day^−1^); “raw/cooked pulses”: class A (<57 g day^−1^) class B (≥57 g day^−1^). For the variables “eggs”, “milk and yogurt from animal sources”, “milk from vegetable sources and its derivatives”, “cheese”, “red meat”, “white meat”, “preserved meat”, and “fish and sea food”, the two classes included volunteers that eat (class YES) or do not eat (class NO) that food category. In round brackets the number of volunteers within each category is reported; * *p* < 0.050.

Figure 2 shows the results of χ^2^ analysis carried out to assess the effects of age and average daily consumption of foods from the arbitrarily defined food categories on the detection frequency of the 12 target genes. No significant differences were seen between males and females (data not shown) as well as between volunteers aged ≥ and <37 years. Moreover, no significant differences were seen between volunteers with low or high daily average consumption of “fruit”, “vegetables”, “pulses”, “eggs”, “milk and yogurt of vegetal origin”, “red meat”, “white meat”, “preserved meat” and “fish and seafood”. When the two food categories “milk and yogurt of animal origin” and “cheese” were considered, significant differences in the occurrence of some AR genes emerged. In more detail, the highest occurrence of *tet*(K) was seen in the saliva of volunteers eating milk and milk-based products from animal sources compared to consumers not eating this specific food category (*p* = 0.0174). Analogously, higher occurrence of *erm*(C) (*p* = 0.0374) and *tet*(K) (*p* = 0.0253) was seen in volunteers regularly consuming cheeses. By contrast, a significantly lower frequency of *tet*(M) (0.0181) was found in the latter volunteers compared to those not eating cheeses.

### 3.4. Principal Component Analysis (PCA)

**Figure 3:**
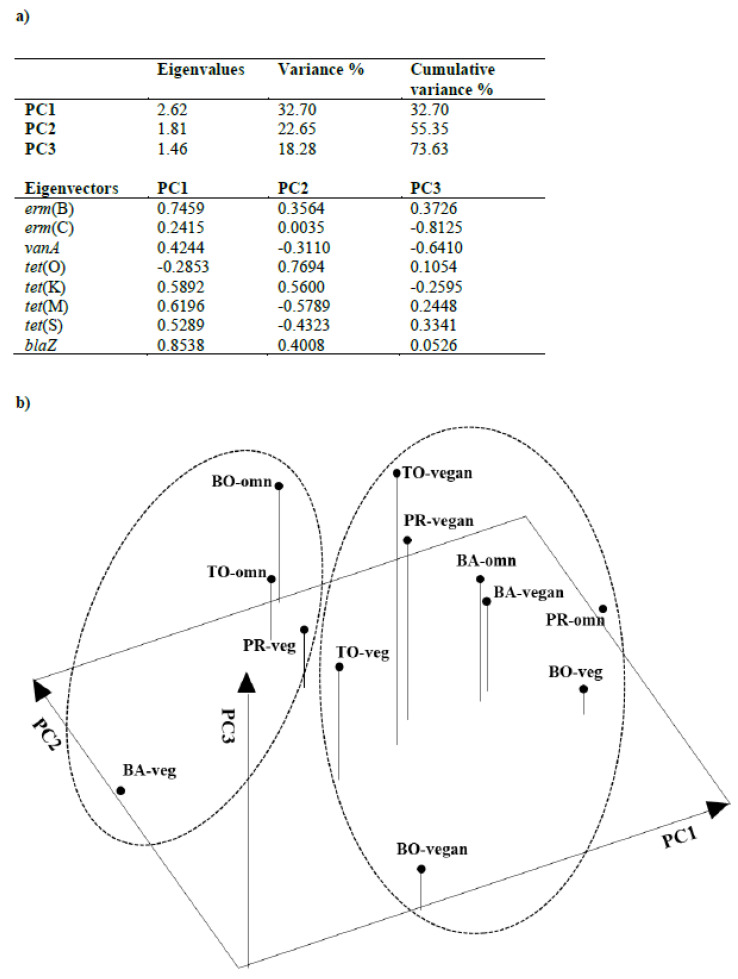
Principal Component Analysis (PCA) results: panel (**a**) eigenvalues, percentage of variance explained and eigenvectors of the principal components; panel (**b**) PCA three-dimensional plot. BA, Bari; BO, Bologna; PR, Parma; TO, Turin; omn, omnivores; veg, ovo-lacto vegetarians.

The following genes were excluded from the PCA analysis: *erm*(A), which was detected in just one saliva sample from a vegan volunteer; *tet*(W), which occurred in all saliva samples analyzed, and *van*B and *mec*A, which were not detected at all. Three principal components (e.g., PC1, PC2 and PC3) explained 73.63% of the total variance (Figure 3a). In more detail, PC1 for the genes *erm*(B), *tet*(K), *tet*(M), *tet*(S) and *blaZ* showed the highest eigenvector coefficients (as absolute values). PC2 showed a contrast between *tet*(O) plus *tet*(K) vs. *tet*(M), whereas *erm*(C) and *vanA* were the most relevant AR genes for PC3.

As shown in the three-dimensional plot (Figure 3b), no clear separation of the volunteers based on either their dietary habits or geographical locations was seen. At Bari, a clear separation of ovo-lacto-vegetarians from vegans and omnivores was seen, which was mainly due to differences in the PC1 scores. At Bologna, the three dietary groups were clearly separated from each other. Ovo-lacto-vegetarians differed from the other two groups in their PC1 scores; in particular, a much higher frequency of *tet*(S) was shown by ovo-lacto-vegetarians than by vegans and omnivores. Omnivores and vegans showed clearly contrasting PC2 scores as the saliva of omnivores showed a much higher frequency of *tet*(O) and *tet*(K) and a slightly lower frequency of *tet*(M) than vegans. At Parma, omnivores differed from vegans and ovo-lacto-vegetarians for PC1, whereas PC2 could not discriminate between the three dietary groups; PC3 separated vegans from omnivores and ovo-lacto-vegetarians. Finally, at Turin, the three dietary groups showed similar PC1 scores, whereas PC2 separated omnivores from vegans and ovo-lacto-vegetarians. Moreover, vegans showed higher PC3 scores than omnivores and ovo-lacto-vegetarians.

Overall, the PCA results did not reveal a clustering trend based on either geographical location or dietary habits; however, differences among the three diets were identified within each recruiting site. Hence, χ^2^ analysis was carried out separately for each geographic location to identify the AR genes responsible for the differences among the three diets within each recruiting site; the PCA tridimensional scatter plot guided the choice for the most interesting orthogonal contrasts to be tested.

For each geographical location, the χ^2^ analysis started from the three x two matrix of absolute frequencies of each AR gene (i.e., three diets x two options, namely, the presence or absence of each AR gene). The two degrees of freedom available allowed two orthogonal contrasts, each with one degree of freedom, to be evaluated; comparisons between diets were made based on the results of the PCA analysis.

For Bari (Table 3, panel A), a significantly lower detection frequency of *erm*(B) and *tet*(S) was seen in the saliva of ovo-lacto-vegetarian volunteers that in the saliva of omnivore and vegan volunteers; this finding agrees well with the results of the PC1 scores (Figure 3a). Moreover, no significant differences were found between omnivores and vegans who were recruited at this geographical site (Table 3, panel B).

Differently from Bari, at Bologna (Table 3, panel C), the saliva of ovo-lacto-vegetarians was characterized by a significantly higher detection frequency of *erm*(C) and *tet*(S) than the saliva of omnivores and vegans. Moreover, a significantly higher detection frequency of *tet*(O) and *tet*(K) was found in omnivores than in vegans (Table 3, panel D).

For Parma and Turin, the PCA results suggested two contrasts, namely, “omnivores vs. ovo-lacto-vegetarians + vegans” and “ovo-lacto-vegetarians vs. vegans”. No differences were found between the three dietary groups recruited at Parma (Table 3, panels E and F), whereas omnivores from Turin (Table 3, panel G) significantly differed from ovo-lacto-vegetarians and vegans from the same site for the higher and lower occurrences of *erm*(C) and *tet*(S), respectively; in addition, the saliva of vegans was characterized by a significantly higher frequency of *tet*(O) than the saliva of ovo-lacto-vegetarians (Table 3, panel H).

Overall, the χ^2^ analysis showed that for *erm*(B), *tet*(S), *erm*(C), *tet*(O), and *tet*(K), significant differences occurred among the three dietary habits with an evident differentiation among the four geographical locations considered.

## 4. Discussion

In an enlightening literature review authored by Rolain [27] that focused on food and the human gut as reservoirs of transferable AR genes, the author suggested that “because the potential pool of AR genes in these environments remains largely unknown, with thousands of AR genes yet to be discovered, future observation of AR gene in these ecosystems is warranted from an ecological perspective”. In the same review, the author suggested to researchers in the AR field the use of both culture and nonculture-based techniques for characterization of the human resistome in response to various conditions. As defined by Wright [35], the resistome refers to the pool of resistance genes within a commensal bacterial population.

The present study took up the challenge launched by Rolain [27] by focusing on the relationships among long-term dietary habits and the distribution of selected transferable AR genes in the human oral cavity of heathy adults using a targeted PCR-based metagenomics approach. The study started from the assumption that due to the broad use of antibiotics in humans, food animals, and agriculture, foods of both animal and plant origin are potential reservoirs of transferable AR genes [27]. This latter hypothesis is largely supported by several literature reviews highlighting the widespread occurrence of AR genes in agro-food chains [19,36] and foodborne microorganisms [37,38]. Very recently, a direct connection between antibiotic use and antibiotic resistance in both humans and food-producing animals was emphasized in a report from the European Food Safety Authority, the European Medicines Agency, and the European Centre for Disease Prevention and Control [39].

As reviewed by Moraes et al. [5], few reports are available in the scientific literature on the occurrence of AR genes in specific oral environments, including saliva, supragingival biofilm, and acute endodontic infections. The importance of characterizing the still poorly explored resistome at the human oral cavity has been very recently pointed out by Carr et al. [30]. Even in this latter study, deriving and comparing the oral and gut resistomes from 788 and 386 shotgun metagenomes, respectively, just 72 saliva samples were analyzed, including just 18 samples from Western Europe. In addition to the scarcity of data on the human saliva resistome, to the authors’ knowledge, no in-depth studies have yet been carried out to investigate the effect of diet and consumption of foods from specific food categories on the distribution of transferable AR genes in the human oral cavity.

In the present investigation, several findings emerged from the analysis of the 144 saliva samples collected from volunteers who were following long-term omnivore, ovo-lacto-vegetarian, or vegan diets and who were recruited at four Italian geographical locations.

Overall, the most common AR gene detected in the oral metagenome was *tet*(W), followed by *erm*(B) and *tet*(M). The ubiquity of the latter two genes in the human oral resistome has previously been revealed by numerous studies analyzing cultivated isolates or genetic material recovered directly from human saliva samples [40,41,42]. Both AR genes were also dominant in the genetic material recovered directly from the feces of the same cohort of subjects considered in this study [29]. The dominance of *tet*(M) and *erm*(B) in the human resistome has previously been explained by their cooccurrence in Tn*916*-like elements [29,43]. This hypothesis is fully supported by the results of a previous study [44] that revealed the stable occurrence of the integrase from *Tn916* in the oral metagenome of Italian, Finnish, French, Norwich, and Scottish healthy subjects who had not received antibiotic therapy in the previous three months by using a microarray capable of detecting 23 *tet* and 10 *erm* genes [44].

*Tet*(W) was detected for the first time by Villedieu et al. [22] in bacteria (including lactic acid bacteria) colonizing the oral cavity of healthy adults as the second most common *tet* gene after *tet*(M). Thereafter, the presence of this determinant has frequently been detected in the oral cavity of healthy subjects from Italy and other European countries [41,42,44,45] as well as in lactobacilli isolated from the saliva of caries-active patients [46]. The same gene was also found in fecal DNA from the same cohort of volunteers herein analyzed [29].

In terms of relative abundance, *tet*(S) was the third most frequent gene; this finding was quite unexpected since, according to other authors, its detection in the oral cavity of healthy subjects is somewhat rare [22,42].

Regarding *tet*(K), it has previously been detected in bacterial isolates from the saliva of 20 heathy subjects in the United Kingdom, though with a frequency of 1% of the 105 tetracycline-resistant isolates screened by PCR [22]. As far as the authors know, no data are currently available on the occurrence of this AR gene in the oral metagenomic DNA of healthy subjects from Italy or other European and non-European countries; indeed, neither the study of Card et al. [44] nor that of Seville et al. [42] included *tet*(K) among the genes screened by microarray analysis. Of note, this AR gene was detected in fecal DNA from the same cohort of subjects herein considered, with frequencies ranging from 27% of the 48 vegan subjects to 54% of the 48 omnivore subjects [29]. The *tet*(K) gene, which encodes a tetracycline efflux protein that functions as a metal-tetracycline/H+ antiporter, is commonly localized on tetracycline-resistance plasmids, e.g., pT181 [47] or pKH1 [48] of *Staphylococcus aureus*, thus suggesting a main contribution of conjugative transfer as a potential primary means of spread of this determinant between bacteria.

As far as *tet*(O) is concerned, the very low recovery of this gene (<0.20 frequency) in the cohort of subjects herein considered agrees well with the low detection of this determinant in the oral microbiome of Norwegian and Finnish subjects, as previously reported by Seville et al. [42]. Interestingly, *tet*(O) was also the least abundant *tet* gene in the feces of the same cohort of volunteers [29]. A completely different picture emerged from earlier studies [22,41], which reported that *tet*(O) was the second most frequently detected *tet* gene in the human oral cavity.

Among the *erm* genes, in addition to the almost ubiquitous *erm*(B), the *erm*(C) and *erm*(A) genes were also detected but with considerably lower frequencies. In a previous study investigating the presence and prevalence of selected AR genes in the metagenomic DNA isolated from the saliva of volunteers from six European countries, including Italy, *erm*(C) was detected only in Norwegian and Scottish samples [42]. No data are available so far on the incidence of *erm*(A) in the human oral cavity; notably, the distribution of this determinant in the cohort of subjects herein considered overlaps with that of the study performed on the feces collected from the same subjects, with only one vegan volunteer carrying this gene [29].

It has been previously hypothesized that the widespread use of tetracyclines and macrolides in the meat and fresh produce industries has led to high occurrences of tetracycline- and erythromycin-resistant bacteria in foods, thus potentially contributing to the transfer and spread of these AR genes to the commensal microbiota of the oral cavity [21,49]. In support of this thesis, according to the ninth European Surveillance of Veterinary Antimicrobial Consumption (ESVAC) Report on trends from 2010 to 2017 for sales of veterinary antimicrobial agents in 29 European Union (EU) and European Economic Area (EEA) countries [50], Italy was among the countries with the highest sales of tetracyclines, macrolides, and penicillin for food-producing animals.

As for β-lactam resistance, *blaZ* was detected in the nearly half of the saliva samples screened in the present study, whereas *mecA* was never detected. In two recent studies, *blaZ* was found to prevail among those *Staphylococcus aureus* strains that were isolated from the oral cavity of patients with periodontitis [51] and Tunisian children [52]. Martinez et al. [28] ranked *blaZ* into RESCon 1 category, which comprises AR genes that are located on mobile genetic elements hosted by human pathogens, thus posing a substantial risk for the distribution of resistance and treatment failure of human infections. Though most authors have reported the prevalence of β-lactam resistance genes in subjects with oral diseases, more recent investigations have highlighted a high prevalence of these determinants in the oral microbiota of healthy subjects [53].

Regarding the vancomycin resistance genes, *vanA* was the sole determinant detected in the present study at a low frequency. This latter finding is not surprising, given the acknowledged association of this resistance gene with the enterococci, a group of microorganisms considered as temporary components of the oral microbiota [54]. Contaminated and even fermented foods have previously been hypothesized as the main sources of vancomycin-resistant enterococci in the oral cavity [55]. Thus far, the transfer of vancomycin resistance genes between different strains of *Enterococcus faecium*, *Enterococcus faecalis*, and *S. aureus* has been reported [40].

When the overall effect of diet on the occurrence of the 12 target genes was evaluated by PCA, no clear separations were seen between the three dietary groups. This finding agrees very well with the results of the 16S rRNA gene pyrosequencing of the DNA extracted from the same saliva samples herein analyzed [10] which clearly revealed a stable core of bacterial taxa that was not influenced by the dietary habits of the volunteers. Nevertheless, in the latter investigation [10], diet-related biomarkers which distinguished omnivore vs. non omnivore volunteers were identified, thus supporting what was previously reported by Takeda et al. [56] on the contribution of environmental factors in the modulation of salivary metabolomics profiles. In a more recent study, the increased consumption of plant foods through a Mediterranean diet caused a decrease in salivary levels of potential periodontopathogenic bacteria [11].

In addition, no significant clustering of saliva samples was revealed by PCA based on the recruiting site. Hence, further analyses were carried out to explore the occurrence of significant differences among the three dietary groups at each recruiting site, separately. This further elaboration allowed some significant differences to be highlighted, such as the higher frequency of *erm*(C) and *tet*(S) in the saliva of ovo-lacto-vegetarians than in the saliva of vegans and omnivores from Bologna or the higher abundance of *tet*(O) and *tet*(K) in omnivores than in vegans from the same recruiting site. Significant differences were also found among the volunteers recruited in Turin, with *erm*(C) and *tet*(S) being detected with a significantly higher frequency in omnivores than in ovo-lacto-vegetarians and vegans and *tet*(O) being significantly more abundant in vegans than in ovo-lacto-vegetarians.

A reasonable interpretation of the latter findings is cumbersome, and some evident contradictions emerge, such as the considerably higher frequency of *tet*(O) in saliva samples collected from volunteers following two opposite dietary habits, namely, vegans from Turin and omnivores from Bologna. On the other hand, the higher occurrence of *erm*(C) in omnivores from Turin and ovo-lacto-vegetarians from Bologna agrees well with the higher occurrence of *tet*(K) in omnivores from the latter recruiting site, with both genes being positively correlated with the consumption of cheese and dairy products, as revealed by χ^2^ tests. A similar association between *tet*(K) and the consumption of dairy products emerged from the analysis of the metagenomic DNA isolated from the feces of the same cohort herein considered [29]. To date, different authors have reported the isolation of coagulase-negative staphylococci carrying *tet*(K) from raw milk and Italian cheeses [57,58]. In a past study, the same gene was successfully quantified by qPCR in both Italian and Spanish retail cheeses [59], whereas *erm*(C) was detected in lactic acid bacteria isolated from Italian water buffalo Mozzarella cheese and in the raw milk and natural whey starters used for its manufacture [60]. The *erm*(C) gene was also found in samples of artisan cheese manufactured in Poland with unpasteurized cow milk [61], while Schlegelova et al. [62] indicated that the dairy environment, including the surfaces of milking and processing equipment for cheese manufacturing, was a potential source of *erm*(C).

In addition to the effects of diet evident only at the recruitment site level and correlation of some specific food categories with the occurrence of a few AR genes, personal oral hygiene, potable water, and some other unascertained factors may influence AR gene distribution in human saliva. Among the latter factors, personal history of acute odontogenic infections and periodontitis, requiring treatment with antibiotics, must be included. Concerning this latter issue, β-lactams, macrolides, and tetracylines are among the antibiotics most frequently prescribed by dentists for dental health [63].

Since the spread of AR genes in natural ecosystems can challenge the dynamics and physiology of natural microbial populations, AR genes may be considered environmental pollutants [64]. Consequently, sufficiently distant geographical areas such as those considered in our study for recruiting volunteers will probably be characterized by different bacteria that carry different AR determinants and that may spread farther along local food chains through direct or indirect contact.

Finally, no effect of age or sex was seen on the occurrence of the 12 AR genes under study, thus confirming what was previously found by analyzing the feces collected from the same cohort of subjects herein considered [29].

## 5. Conclusions

No indication that a specific long-term diet such as omnivore, ovo-lacto-vegetarian or vegan could significantly impact the abundance of the AR genes in human saliva emerged from the present study. No effects of either age or sex were seen. Some differences among the three diets emerged only when each recruiting site was considered independently. Otherwise, regular consumption of specific food categories such as milk and milk-based products, including cheese, could lead to a significant increase in genes encoding resistance to tetracycline and erythromycin. Undoubtedly, fermented foods including milk-based ones, such as cheese and yogurt, represent important vehicles for the entrance of high numbers of living bacteria potentially carrying AR genes into the human oral cavity and gastrointestinal tract. Therefore, limited use of antibiotics in farming of milk animals together with hygiene improvement and control in dairy processing may contribute to the prevention of the spread of AR genes in this food category.

Overall, the high prevalence of some AR genes in the saliva of the large cohort of volunteers considered in the present study, independent of their dietary habits, recruiting site, age, or sex, suggests widespread occurrence of transferable resistances in the human oral cavity reservoir. A feasible explanation of this finding might be the reliance on the extensive use of antibiotics in livestock production, aquaculture, and crop culture, which in turn explains the highly documented detection of transferable AR genes in agro-food chains and foodborne microorganisms thus far.

According to the recently published Global Action Plan on Antimicrobial Resistance [65], surveillance and monitoring of AR genes are among the key objectives for minimizing the impact of antibiotic resistance. Hence, further research efforts are needed to better elucidate the effects of either dietary habits or consumption of specific food categories on the human saliva resistome.

## Figures and Tables

**Table 1:** The mean quantities (g or mL day^−1^) of foods with potentially high risk for the introduction of antibiotic resistance (AR) microorganisms and their genes into the human oral cavity regularly consumed by vegans, ovo-lacto vegetarians and omnivores.

Food Categories (g or mL day^−1^)	Diet		
	Vegans	Ovo-Lacto Vegetarians	Omnivores
Fruits	689.7 ± 983.1	303.7 ± 194.4	181.3 ± 108.0
Vegetables	520.8 ± 210.8	438.1 ± 189.0	270.1 ± 107.5
Pulses	115.2 ± 75.5	92.4 ± 55.3	16.0 ± 26.6
Milk from vegetable sources and its derivatives	135.1 ± 113.7	74.7 ± 89.1	8.5 ± 43.4
Eggs	n.c.	15.8 ± 15.4	15.0 ± 12.1
Milk and yogurt from animal sources	n.c.	60.2 ± 94.0	137.6 ± 140.5
Cheese	n.c.	49.2 ± 39.1	61.3 ± 32.3
Red meat	n.c.	n.c.	35.3 ± 31.9
White meat	n.c.	n.c.	39.9 ± 43.7
Preserved meat	n.c.	n.c.	46.3 ± 26.5
Fish and seafood	n.c.	n.c.	38.9 ± 31.2

Data are means ± standard deviation; n.c., not consumed.

**Table 2:** Positive results of PCR and nested PCR (n-PCR) assays targeting the 12 antibiotic resistance (AR) genes of interest, carried out onto saliva samples collected from the three groups of heathy volunteers (each including 48 individuals) following long-term (>1 year) omnivore, ovo-lacto-vegetarian or vegan diet.

Diet (Number of Volunteers)	Assay	Number of Positive Samples											
		*erm*(A)	*erm*(B)	*erm*(C)	*vanA*	*vanB*	*tet*(O)	*tet*(M)	*tet*(W)	*tet*(S)	*tet*(K)	*mecA*	*blaZ*
Omnivore (48)	PCR	0	31	5	0	0	0	44	46	1	11	0	15
	n-PCR	0	17	9	5	0	9	0	2	19	13	0	13
	**Total**	**0**	**48**	**14**	**5**	**0**	**9**	**44**	**48**	**20**	**24**	**0**	**28**
Vegan (48)	PCR	0	35	1	0	0	0	47	48	0	7	0	11
	n-PCR	1	12	7	5	0	6	1	n.a.	26	8	0	10
	**Total**	**1**	**47**	**8**	**5**	**0**	**6**	**48**	**48**	**26**	**15**	**0**	**21**
Ovo-lacto vegetarian (48)	PCR	0	23	0	0	0	0	43	45	0	8	0	13
	n-PCR	0	22	17	5	0	7	0	3	24	11	0	6
	**Total**	**0**	**45**	**17**	**5**	**0**	**7**	**43**	**48**	**24**	**19**	**0**	**19**
Whole cohort (144)	PCR	0	89	6	0	0	0	134	139	1	26	0	39
	n-PCR	1	51	33	15	0	22	1	5	69	32	0	29
	**Total**	**1**	**140**	**39**	**15**	**0**	**22**	**135**	**144**	**70**	**58**	**0**	**68**

n.a., not applicable.

**Table 3:** Results of orthogonal contrasts between dietary habits within each recruiting site.

**(A) Bari**				**(B) Bari**			
**AR gene**	**Ovo-lacto vegetarians** **(*n* = 12)**	**Omnivores + Vegans** **(*n* = 27)**	***p***	**AR gene**	**Omnivores** **(*n* = 14)**	**Vegans** **(*n* = 13)**	***p***
*erm*(B)	10 (83%)	27 (100%)	0.026 *	*erm*(B)	14 (100%)	13 (100%)	n.a.
*erm*(C)	4 (33%)	8 (30%)	0.818	*erm*(C)	4 (29%)	4 (31%)	0.901
*vanA*	1 (8%)	3 (11%)	0.788	*vanA*	1 (7%)	2 (15%)	0.493
*tet*(O)	3 (25%)	3 (11%)	0.283	*tet*(O)	1 (7%)	2 (15%)	0.493
*tet*(K)	4 (33%)	12 (44%)	0.512	*tet*(K)	6 (43%)	6 (46%)	0.863
*tet*(M)	10 (%)	26 (96%)	0.182	*tet*(M)	13 (93%)	13 (100%)	0.245
*tet*(S)	3 (25%)	17 (63%)	0.026 *	*tet*(S)	10 (71%)	7 (54%)	0.343
*blaZ*	3 (25%)	15 (56%)	0.072	*blaZ*	8 (57%)	7 (54%)	0.863
**(C) Bologna**				**(D) Bologna**			
**AR gene**	**Ovo-lacto vegetarians** **(*n* = 12)**	**Omnivores + Vegans** **(*n* = 24)**	***p***	**AR gene**	**Omnivores** **(*n* = 13)**	**Vegans** **(*n* = 11)**	***p***
*erm*(B)	12 (100%)	23 (96%)	0.364	*erm*(B)	13 (100%)	10 (91%)	0.204
*erm*(C)	7 (58%)	5 (21%)	0.026 *	*erm*(C)	2 (15%)	3 (27%)	0.475
*vanA*	2 (17%)	4 (17%)	1.000	*vanA*	1 (8%)	3 (27%)	0.194
*tet*(O)	1 (8%)	4 (17%)	0.479	*tet*(O)	4 (31%)	0 (0%)	0.018 *
*tet*(K)	6 (50%)	8 (33%)	0.336	*tet*(K)	7 (54%)	1 (9%)	0.015 *
*tet*(M)	12 (100%)	22 (92%)	0.195	*tet*(M)	11 (85%)	11 (100%)	0.107
*tet*(S)	10 (83%)	10 (42%)	0.014 *	*tet*(S)	5 (38%)	5 (45%)	0.729
*blaZ*	6 (50%)	10 (42%)	0.636	*blaZ*	7 (54%)	3 (27%)	0.184
**(E) Parma**				**(F) Parma**			
**AR gene**	**Ovo-lacto vegetarians + Vegans** **(*n* = 24)**	**Omnivores** **(*n* = 9)**	***p***	**AR gene**	**Vegans** **(*n* = 12)**	**Ovo-lacto vegetarians** **(*n* = 12)**	***p***
*erm*(B)	24 (100%)	9 (100%)	n.a.	*erm*(B)	12 (%)	12	n.a.
*erm*(C)	5 (21%)	3 (33%)	0.465	*erm*(C)	1 (8%)	4 (33%)	0.121
*vanA*	2 (8%)	3 (33%)	0.092	*vanA*	0 (0%)	2 (17%)	0.086
*tet*(O)	4 (17%)	1 (11%)	0.684	*tet*(O)	1 (8%)	3 (25%)	0.264
*tet*(K)	10 (42%)	6 (67%)	0.198	*tet*(K)	6 (50%)	4 (33%)	0.406
*tet*(M)	22 (92%)	9 (100%)	0.250	*tet*(M)	12 (100%)	10 (83%)	0.086
*tet*(S)	11 (46%)	4 (44%)	0.943	*tet*(S)	6 (50%)	5 (42%)	0.682
*blaZ*	10 (42%)	7 (78%)	0.058	*blaZ*	5 (42%)	5 (42%)	1.000
**(G) Turin**				**(H) Turin**			
**AR gene**	**Ovo-lacto vegetarians +Vegans** **(*n* = 24)**	**Omnivores** **(*n* = 12)**	***p***	**AR gene**	**Vegans** **(*n* = 12)**	**Ovo-lacto vegetarians** **(*n* = 12)**	***p***
*erm*(B)	23 (96%)	12 (100%)	0.364	*erm*(B)	12 (100%)	11 (92%)	0.232
*erm*(C)	2 (8%)	5 (42%)	0.020 *	*erm*(C)	0 (0%)	2 (17%)	0.086
*vanA*	0 (0%)	0 (0%)	n.a.	*vanA*	0 (0%)	0 (0%)	n.a.
*tet*(O)	3 (12%)	3 (25%)	0.354	*tet*(O)	3 (25%)	0 (0%)	0.032 *
*tet*(K)	7 (29%)	5 (42%)	0.457	*tet*(K)	2 (17%)	5 (42%)	0.173
*tet*(M)	23 (96%)	11 (92%)	0.617	*tet*(M)	12 (100%)	11 (92%)	0.232
*tet*(S)	14 (58%)	1 (8%)	0.002 *	*tet*(S)	5 (42%)	9 (75%)	0.094
*blaZ*	11 (46%)	6 (50%)	0.813	*blaZ*	5 (42%)	6 (50%)	0.682

n, number of volunteers; n.a., not applicable; *, significantly different.

## Supplementary Materials

The following are available online at https://www.mdpi.com/2073-4425/11/9/1088/s1, Table S1: The list of vegans, ovo-lacto vegetarians and omnivores included in study, each identified with an anonymous code, age, sex and geographical origin.
