# Supplementary material for: Distribution of Antibiotic Resistance Genes in the Saliva of Healthy Omnivores, Ovo-Lacto-Vegetarians, and Vegans
Source: Genes (Basel). 2020 Sep 18;11(9):1088. doi: 10.3390/genes11091088 (PMC7564780; doi:10.3390/genes11091088)
Supplement: Supplementary file 1 [file genes-11-01088-s001.pdf]

**Supplementary Table 1.** The list of vegans, ovo-lacto vegetarians and omnivores included in study, each identified with an anonymous code, age, sex and geographical origin.

| Subject identification code | Diet | Sex | Age | Geographical location |
|---|---|---|---|---|
| 01TO | ovo-lacto vegetarian | F | 30 | Turin |
| 02TO | ovo-lacto vegetarian | F | 42 | Turin |
| 03TO | vegan | M | 46 | Turin |
| 04TO | ovo-lacto vegetarian | M | 53 | Turin |
| 06TO | ovo-lacto vegetarian | M | 43 | Turin |
| 07TO | vegan | M | 42 | Turin |
| 10TO | vegan | F | 30 | Turin |
| 11TO | vegan | M | 38 | Turin |
| 13TO | omnivore | F | 47 | Turin |
| 14TO | omnivore | F | 48 | Turin |
| 15TO | omnivore | M | 31 | Turin |
| 16TO | omnivore | F | 39 | Turin |
| 17TO | omnivore | F | 47 | Turin |
| 18TO | omnivore | F | 39 | Turin |
| 19TO | vegan | M | 48 | Turin |
| 21TO | vegan | F | 59 | Turin |
| 22TO | omnivore | M | 31 | Turin |
| 23TO | omnivore | F | 39 | Turin |
| 24TO | vegan | F | 37 | Turin |
| 25TO | vegan | F | 50 | Turin |
| 26TO | ovo-lacto vegetarian | M | 52 | Turin |
| 27TO | vegan | M | 37 | Turin |
| 28TO | ovo-lacto vegetarian | F | 53 | Turin |
| 29TO | ovo-lacto vegetarian | F | 48 | Turin |
| 31TO | omnivore | M | 39 | Turin |
| 32TO | vegan | F | 35 | Turin |
| 33TO | omnivore | F | 45 | Turin |
| 34TO | ovo-lacto vegetarian | M | 35 | Turin |
| 35TO | vegan | F | 35 | Turin |
| 37TO | omnivore | F | 39 | Turin |
| 38TO | ovo-lacto vegetarian | M | 47 | Turin |
| 39TO | ovo-lacto vegetarian | F | 37 | Turin |
| 41TO | ovo-lacto vegetarian | F | 32 | Turin |
| 42TO | ovo-lacto vegetarian | M | 38 | Turin |
| 43TO | omnivore | F | 43 | Turin |
| 44TO | vegan | M | 44 | Turin |
| 01BA | ovo-lacto vegetarian | M | 33 | Bari |
| 02BA | ovo-lacto vegetarian | F | 28 | Bari |
| 03BA | ovo-lacto vegetarian | M | 40 | Bari |
| 04BA | ovo-lacto vegetarian | F | 28 | Bari |
| 05BA | ovo-lacto vegetarian | F | 43 | Bari |
| 07BA | ovo-lacto vegetarian | F | 30 | Bari |
| 08BA | ovo-lacto vegetarian | F | 55 | Bari |
| 09BA | ovo-lacto vegetarian | F | 30 | Bari |
| 10BA | ovo-lacto vegetarian | M | 28 | Bari |
| 11BA | ovo-lacto vegetarian | F | 28 | Bari |
| 12BA | ovo-lacto vegetarian | F | 55 | Bari |
| 13BA | ovo-lacto vegetarian | F | 27 | Bari |
| 14BA | vegan | M | 52 | Bari |
| 15BA | vegan | F | 29 | Bari |
| 16BA | vegan | M | 28 | Bari |
| 17BA | vegan | F | 30 | Bari |
| 18BA | vegan | M | 50 | Bari |
| 19BA | vegan | F | 25 | Bari |
| 20BA | vegan | M | 28 | Bari |

| | | | | |
|---|---|---|---|---|
| 21BA | vegan | F | 28 | Bari |
| 22BA | vegan | M | 40 | Bari |
| 23BA | vegan | F | 25 | Bari |
| 24BA | vegan | F | 37 | Bari |
| 25BA | vegan | F | 40 | Bari |
| 26BA | vegan | F | 25 | Bari |
| 28BA | omnivore | F | 30 | Bari |
| 29BA | omnivore | M | 32 | Bari |
| 30BA | omnivore | M | 35 | Bari |
| 31BA | omnivore | F | 34 | Bari |
| 32BA | omnivore | M | 37 | Bari |
| 33BA | omnivore | F | 32 | Bari |
| 34BA | omnivore | M | 38 | Bari |
| 35BA | omnivore | F | 24 | Bari |
| 36BA | omnivore | F | 30 | Bari |
| 37BA | omnivore | M | 28 | Bari |
| 38BA | omnivore | F | 27 | Bari |
| 39BA | omnivore | M | 28 | Bari |
| 40BA | omnivore | M | 25 | Bari |
| 41BA | omnivore | F | 28 | Bari |
| 01BO | ovo-lacto vegetarian | F | 50 | Bologna |
| 02BO | vegan | F | 28 | Bologna |
| 03BO | omnivore | M | 53 | Bologna |
| 04BO | ovo-lacto vegetarian | F | 45 | Bologna |
| 05BO | ovo-lacto vegetarian | F | 42 | Bologna |
| 06BO | omnivore | M | 37 | Bologna |
| 07BO | vegan | F | 30 | Bologna |
| 08BO | omnivore | M | 48 | Bologna |
| 09BO | omnivore | M | 48 | Bologna |
| 10BO | vegan | F | 44 | Bologna |
| 11BO | vegan | F | 30 | Bologna |
| 12BO | vegan | M | 43 | Bologna |
| 13BO | vegan | M | 45 | Bologna |
| 16BO | omnivore | F | 29 | Bologna |
| 17BO | ovo-lacto vegetarian | M | 26 | Bologna |
| 18BO | vegan | M | 33 | Bologna |
| 19BO | ovo-lacto vegetarian | M | 37 | Bologna |
| 20BO | omnivore | F | 59 | Bologna |
| 22BO | omnivore | F | 34 | Bologna |
| 23BO | omnivore | F | 30 | Bologna |
| 24BO | omnivore | M | 32 | Bologna |
| 25BO | ovo-lacto vegetarian | F | 43 | Bologna |
| 26BO | vegan | F | 32 | Bologna |
| 27BO | ovo-lacto vegetarian | M | 42 | Bologna |
| 28BO | omnivore | F | 21 | Bologna |
| 29BO | omnivore | F | 25 | Bologna |
| 30BO | omnivore | F | 30 | Bologna |
| 32BO | ovo-lacto vegetarian | F | 49 | Bologna |
| 34BO | ovo-lacto vegetarian | F | 40 | Bologna |
| 35BO | vegan | F | 54 | Bologna |
| 36BO | vegan | M | 56 | Bologna |
| 37BO | ovo-lacto vegetarian | F | 32 | Bologna |
| 39BO | ovo-lacto vegetarian | F | 30 | Bologna |
| 40BO | ovo-lacto vegetarian | M | 32 | Bologna |
| 42BO | omnivore | F | 46 | Bologna |
| 44BO | vegan | F | 32 | Bologna |
| 01PR | omnivore | M | 30 | Parma |
| 02PR | ovo-lacto vegetarian | F | 32 | Parma |
| 03PR | ovo-lacto vegetarian | M | 56 | Parma |
| 04PR | ovo-lacto vegetarian | F | 49 | Parma |
| 05PR | ovo-lacto vegetarian | F | 44 | Parma |
| 06PR | ovo-lacto vegetarian | F | 40 | Parma |

| | | | | |
|---|---|---|---|---|
| 07PR | vegan | M | 31 | Parma |
| 08PR | ovo-lacto vegetarian | F | 35 | Parma |
| 09PR | vegan | F | 23 | Parma |
| 10PR | ovo-lacto vegetarian | M | 47 | Parma |
| 11PR | vegan | M | 26 | Parma |
| 12PR | vegan | F | 41 | Parma |
| 13PR | ovo-lacto vegetarian | F | 31 | Parma |
| 14PR | ovo-lacto vegetarian | M | 36 | Parma |
| 15PR | ovo-lacto vegetarian | F | 18 | Parma |
| 17PR | vegan | F | 40 | Parma |
| 21PR | vegan | M | 35 | Parma |
| 22PR | vegan | M | 30 | Parma |
| 23PR | vegan | M | 41 | Parma |
| 25PR | omnivore | F | 48 | Parma |
| 26PR | vegan | M | 25 | Parma |
| 27PR | ovo-lacto vegetarian | F | 31 | Parma |
| 29PR | omnivore | F | 31 | Parma |
| 30PR | ovo-lacto vegetarian | M | 23 | Parma |
| 31PR | omnivore | M | 35 | Parma |
| 32PR | vegan | F | 39 | Parma |
| 33PR | vegan | F | 29 | Parma |
| 34PR | omnivore | M | 40 | Parma |
| 35PR | vegan | F | 58 | Parma |
| 36PR | omnivore | F | 36 | Parma |
| 37PR | omnivore | M | 39 | Parma |
| 38PR | omnivore | M | 38 | Parma |
| 39PR | omnivore | M | 38 | Parma |
